# Tears and transformation: feeling like crying as an indicator of insightful or “aesthetic” experience with art

**DOI:** 10.3389/fpsyg.2015.01006

**Published:** 2015-07-23

**Authors:** Matthew Pelowski

**Affiliations:** ^1^Department of Basic Research and Research Methods, Faculty of Psychology, University of ViennaVienna, Austria; ^2^Graduate School of Information Science, Nagoya UniversityNagoya, Japan

**Keywords:** crying, tears, art, insight, schema change, aesthetic experience, cognitive model, museum study

## Abstract

This paper explores a fundamental similarity between cognitive models for crying and conceptions of insight, enlightenment or, in the context of art, “aesthetic experience.” All of which center on a process of initial discrepancy, followed by schema change, and conclude in a proposed adjustment or “transformation” of one's self image/world-view. Because tears are argued to mark one of the only physical indicators of this cognitive outcome, and because the process is particularly salient in examples with art, I argue that crying may provide an intriguing marker for empirical study of art experience. To explore this parallel, I offer a review of crying theory as well as of tearful cases with art, pointing out the key cognitive elements. I then introduce an expanded crying model, based upon our recent model of art experience which does consider insight and adjustment or application of the self. I also consider multiple emotional and evaluative factors, which may co-vary with crying response. This theoretical discussion is then applied in three exploratory, survey-based studies conducted within U.K., Japan and U.S. museums, and including what is claimed to be the 20th century's most tear-inducing abstract paintings. Results showed—with cross-cultural consistency—significant relation between “feeling like crying” and a collection of responses posited to indicate a full progression to aesthetic experience, as well as to positive assessment of artwork goodness, beauty, understanding of meaning, and to final reported self reflection and epiphany. I argue that, beyond the question of *why* we may cry, by considering the implications of *what* tears may indicate within information processing, feeling like crying may indeed offer a compelling basis for empirically identifying outcomes of perceptual (art) experience.

## Introduction

The phenomenon of tears in perceptual experience has long been intriguing. In anthropology, Freedberg (1989) argues that tears, though rare, are among the most universal responses to visual images. Lutz (1999) gives a similar claim for tears in sociology. Add the (often anecdotal) evidence for what types of stimuli evoke crying, and this topic becomes even more compelling for psychology. While a great deal of crying research (Vingerhoets et al., 1997 for review) has focused on interpersonal relations, it is often accounts of inanimate and “simple” things—a sunset, a sculpture, or painting—that stand out in popular discussion (e.g., Frey and Langseth, 1985; Lutz, 1999). Individuals cry before stimuli that do not change. They cry before stimuli that are not sad, or those which are beautiful, empty or over-/under-whelming. This may be nowhere more pronounced than within the domain of art, which serves as a nexus for such encounters (see Elkins, 2001 for review). Yet, despite much work of a belletristic nature, there has been little effort to address art- or image-related crying from a psychological or empirical perspective (Wolterstorff, 2003). “Why do we cry” in these cases of perception, Lutz (p. 19) concluded in a recent review, “what, exactly, do tears express?”

It may be this latter question, however, that holds the greatest potential, particularly for psychological study of art. This paper explores a novel approach to crying, based on a cross-topic theoretical review of current theory regarding crying response. This poses a two-factor model, which suggests that tears arise from a cognitive/physiological mechanism involving initial discrepancy in interaction and a second change in schema/expectations that allows for resolution and which can theoretically be connected to an adjustment or “transformation” in the structure of the self. This model has made an important contribution in uniting myriad discussions of crying, integrating behavioral, cognitive and physiological elements. Most intriguing for the present study, when the model is explored for its theoretical basis, the outcome and cognitive progression posited for crying appear to share a fundamental overlap with recent discussion of insight and “aesthetic” experience with art. Thus, beyond the question of *why* we may cry, by considering implications of *what* tears may indicate for information processing, feeling like crying may be a useful tool in analysis of perception or viewing art.

I will consider the above parallels with the goal of providing a platform for future theoretical and empirical research. I review the crying theory and its emphasis on cognitive schema change, connecting this to examples of crying with art. Based on our previous model of art viewing, I then propose an updated model of the key crying progression which does explicitly consider the implications of schema change. I also consider a number of other hypothesized emotional, evaluative, self-referential, and understanding factors posited to connect to the model stages and which may show empirical correlation with a crying response. I also consider efficacy of using self-report “feeling like crying,” as opposed to direct physiological measure, as an unobtrusive means of recording tearful insight. I conclude by applying this model to three exploratory studies of feeling like crying, conducted with museum art.

## Review: the two-factor theory of crying and self/schema change

The last quarter century has seen an emerging consensus regarding the source of adult (i.e., non-infantile or chemically-induced Nelson, 1998) crying. This marks an important change from earlier approaches. Labeled by Labott and Martin (1988) as “the hydraulic overflow theory,” previous theories, both in psychology (Borgquist, 1906; Bindra, 1972) and philosophy (Koestler, 1964; Darwin, 1965), had looked for specific emotional or contextual elements and then attempted to sort responses into varieties of “crying situations.” The cognitive approach on the other hand, introduced by Efran and Spangler (1979; refined by Frey and Langseth, 1985; Labott and Martin, 1988; Vingerhoets et al., 1997; Miceli and Castelfranchi, 2003), argues that the pertinent element is not whether one felt happy or sad, but rather a specific outcome of information processing centering on two key events: (1) an initial arousal of cognitive dissonance due to discrepancy between expectations and perception, leading to a period of tension or anxiety in which individuals search for resolution or try to repress discrepancy's consequence; followed by (2) either a psychological or external “triggering” change that causes re-assessment and elicitation of tears.

To take one example, the movie *Brian's Song* (ABC TV Movie of the week; Witt and Kulik, 1971), which has served as a reliably tear-evoking stimulus (Frey and Langseth, 1985): a viewer, presented with a story of a young well-liked football player, forms an initial hopeful expectation (1A). The character is then stricken with chronic illness (1B), with which both character and viewer spend the majority of the movie attempting to cope; until a final development—the death of the character (2)—causes one to give up expectations, accept reality and, only after these two events, to potentially begin crying.

While on the surface this seems a rather simplistic explanation—one would naturally expect crying at a character's death—it is this progression that is argued to unite all crying cases. More importantly, when looking at the cognitive underpinnings of this process, prevalent discussion grounds the crying outcome in a specific mechanism whereby viewers confront discrepancies within the act of information processing in terms of relation to their self. Discrepancies can arise for any number of reasons—failure, loss (Frey and Langseth, 1985), interpersonal tensions (Frey et al., 1983), confusion, difficulty (Efran and Spangler, 1979), even empathically through fictitious events (Labott and Martin, 1988). In each case, discrepancy involves two relative cognitions, representing a viewer's hypothetical image of the world or perceptual and/or conceptual schema and the perceived “reality” of a given moment. This is experienced as a threat to the self, challenging existing schema (Hill and Martin, 1997) and leading to sympathetic nervous system (fight/flight) response (Patel, 1993; Gross et al., 1994). Discrepancy may also be sad. However, in numerous studies (Labott and Martin, 1988 for review) in which a viewer is presented with a first arousing or discrepant portion of this sequence, while withholding final (either sad or happy) resolution, there is an inverse correlation with crying. Rather, the key point comes to be the second event, regarding how an individual responds.

Because this involves schema- or self-questioning, individuals typically structure interactions to avoid such discrepant events, employing pre-expectational filters, or, if discrepancy arises, attempting to diminish or escape from involvement (e.g., Pelowski and Akiba, 2011). Crying, on the other hand, is argued to occur specifically in those rare cases when viewers do not assimilate or escape. Forced into an intractable position, they instead adopt a self-referential approach to interaction, acknowledging discrepancy, “giving up” attempts at overt control, eventually confronting and changing their own schema or expectations, and thereby through such schema change, causing the previous “psychological barrier or perturbation to disappear” (Efran and Spangler, 1979, p. 63).

This then becomes the crux of crying. Crying arousal is argued to be a physiological response of the parasympathetic nervous system accompanying relief of tension following schema change (Frey and Langseth, 1985; Patel, 1993; Gross et al., 1994). And tears, placed within this cognitive frame, become a specific “sign” for this “cognitive or psychological reorganization” (Efran and Spangler, p. 68; see also Hill and Martin, 1997); indicating, instead of preservation of existing schema through assimilation or escape, the overcoming of self-protection (Bohart, 1980); coinciding, through underlying tie of schema to the self, with relative change in the self image (Hill and Martin); ultimately signaling what Barbalet (2005) called a “self transformation.” Individuals may report any number of emotions at points during which barriers are presented and embellished, Efran and Spangler (p. 70) conclude, but potentially in all cases (Labott and Martin, 1988; Hendriks and Vingerhoets, 2002 for reviews) when individuals do cry, they report tearfulness “only” at this point where they experience disappearance of a barrier, solution to a problem, or transformation of a previously held worldview^1^.

### The two-factor theory and examples with art

It is here that compelling connection can be made to psychological study of perception and to the unique case of art. First, this two-factor progression and final outcome is readily applicable to especially art interactions. While existing discussion has focused primarily on social and temporal events—where one stumbles across a discrepant life element, attempts to repress or cope with its implication, and eventually gives up or gives in to a changing environment—art-history gives a wealth of examples, in multiple media, of discrepancy, reflection, schema revision, as well as explicit mention of transformations of the self. Several are collected in Table 1, dividing discussion into the two key cognitive elements.

**Table 1:** **Cases of tears from art, considered via the two-factor theory of crying^a^**.

	**Expectation + discrepancy**	**Self/schema transformation**
Abstract/modern painting	Elkins (2001), in a historical review, contends that tears occur when a viewer has a vested interest in perceiving/understanding an artwork yet they find that they cannot understand. He argued interaction “converge(s) on two kinds of experience.” Works seem “unbearably full, complex, daunting, or somehow too close to be properly seen” or “empty, dark, painfully vast, cold, and somehow too far away to be understood” (p. pxi). This leads to uncomfortable “turning point” (p. 11), “the moment when they have seen everything they can, and they sense its time to look away.”	Viewers may either walk away (the abortive outcome in this paper's model) or confront expectations, give up attempt at processing and eventually accept or retune themselves to see the artwork in a new way (coinciding with crying).
Romantic painting	Montgomery (2003; also Miller, 1974) related the case of Caspar David Friedrich, who played with 18th c. expectation of paintings as “picture windows” which viewers can “step into,” instead blocking the gaze with a hill or depicting meaningless voids. In a famous account, Heinrich von Kleist, faced with a painting of a monk staring out to sea (see Figure 1), reported finding himself left “with nothing but a frame as foreground,” and only “infinity” to gaze upon (p. 58).	This forced a reconsideration of his expectations for his typical way of addressing art, causing him to reporting that he felt as if his “eyelids had been cut away” (p. 58).
Baroque/renaissance painting	Magherini (1989; see also Elkins, 2001), considered several cases of discrepancy from depicted mimetic content which question the self. She related a tearful interaction between a “repressed” gay male viewer and Rubens painting of a young man which caused a troubling arousal. This caused a period of anxiety where he repeatedly revisited the work attempting to reconcile or understand his reaction.	Tears arose, according to this review, when the man did experience a realization of his previously denied sexual self. *As* Magherini (in Elkins, p. 47) concluded, *while “different people have strong reactions to the same works of art,” response ultimately comes down to “the [viewer's] history” and subsequent adjustment*.
Post-modern sculpture	Fraser (2006, p. 42), documented her experience with works of Fred Sandback, composed of string strung to make the contours of geometric shapes demarcating ephemeral space. The works, she noted, represented such an “extremely reduced kind of art, art so devoid of anything that would normally be considered expressive and affective” (p. 34), that they led her to question art's very existence. “For me, art [became] an impossibility. And if art is impossible, then artists are also impossible, and I myself am impossible” (p. 42).	She reported crying as result of a final revelation and change in herself. “Just as art cannot exist outside of the field of art, I cannot exist outside of the field of art, *at least not as what I am*, which is an artist” (p. 42). She concluded (p. 34), we cry because we do “not see the person or the object” which had been expected and “must gradually accept the fact that [our] looking is in vain.” She also questioned, would such an outcome be “an aesthetic experience?”
Opera	Poizat (1992) argued that tears arise for a listener when a singer attempts to stretch a note past the range of human ability. The listener “dimly sense[s] that the singer is trying to free themselves from the prison of words,” and they hold (unrealistic) hope/expectation that the note can be sustained.	Tears arise “when a voice has almost made it, but gives out” (p. 146) and the listener finds himself or herself alone in silence, forced to reflect/give up.
Literature	Moretti (1983; also Neale, 1986) argues that crying results from a plot progression in which a reader has more information than a literary character. The reader both identifies with character point of view, representing a naïve/ideal world, while perceiving its untruth. The reader experiences anxiety as the character moves closer to discovery and therefore forcing loss of these expectations.	The reader cries at that point when a “sentence modifies the point of view that had directed our reading,” forcibly “*[re]organizing expectations”* (p. 159).
Music	Sloboda (1985, 1991) argued that tears are caused by “creations and violations of expectancy…within musical structure” (1991, p. 120). He continues, tears are “reliably evoked by melodic appoggiaturas, or grace notes, in which a note above or below the main tone precedes it,” causing a listener “to anticipate an impending ending” (in Lutz, 1999, p. 258), and “creating a certain amount of tension” (1991, p. 120). For example, “the opening six bars of the 3rd movement” of Rachmaninov's Second Symphony is “a prototypical…‘tears’ passage” (1991, p. 115).	Crying then occurs “when the main tone is sounded” forcing either release of expectation that the tone might not sound (in happy/relief cases) or change/giving up if expectation is violated (sad music) (1991, p. 120).
Theater	Brooks (1976; see also Goffman, 1974 for discussion in public galleries) gave an account of tears caused by the experience of an overwhelming emotion or reaction to performance, and which gives one the urge to yell or gesture. However, because one can only remain silent there is created a “fault or gap” between expectations for response and ability to convey emotion (p. 67).	Tears arise when one admits their inability of responding or control. Frijda (1988, p. 351) notes, even in these cases, when tears arise, it is from a switch to “awareness of [one's own] state of action readiness” and ultimately “*some change”* in expectations.
Beauty, perfection, overwhelming/sublime stimuli (1)	Even though such reactions are often tied to harmony or moments of pleasure/being overwhelmed, here there is still discussion of discrepancy. Miceli and Castelfranchi (2003, p. 260) note viewers, faced with a particularly profound or too perfect stimulus, may experience a “sense of contrast between one's experience…and one's [own] perceived ‘smallness’, ordinariness, and imperfection…together with one's helpless need to comprehend and express the whole.” Elkins (2001, p. 20) adds, “viewers see [or experience] more than they expect.” Alternatively, one may again not be able to correctly control their composure or properly respond.	Tears arise when one becomes aware of and acknowledges their own self deficiency or imperfection and gives up the attempt to respond. Koestler (in Lutz, 1999, p. 246) concludes, “there is nothing to be done, no action to be taken, just passive surrender.” *These experiences, Koestler* [in Frey and Langseth, 1985, p. 92] *concludes, are always “self-transcending.”*
Perfection, overwhelming/sublime stimuli (2)	Roald (2009, p. 121) offers another explanation. She claims that such encounters present a self/world just on the horizon of what one previously has conceived and “calls” us to “accept this experience as [our] own.” For example from an interview with a viewer of a sculpture by Barabara Hepworth. “I found it overwhelming…It touched me, and I became agitated…[I was thinking] this is incredible, what is it? What is it with the form?”	Roald argues that by then acknowledging and “accepting” the experience as real, the individual can expand their sense of self to account for the new phenomenon. Ultimately *“the subject calls upon itself to change”* (p. 156). Roald also explicitly linked this event to aesthetic experience.
Happy endings (A)	Miceli and Castelfranchi (2003; also Weiss, 1952) note antecedent discrepancy, claiming “what seems typical of crying for joy is the existence of some prior worry and consequent relief” (p. 257).	Tears tied to final correspondence between reality and expectations, allowed via external changes that allow one to adopt a hoped for schema that had been incongruent.
Happy endings (B)	Alternatively, Neale (1986, p. 21) argues for overt dissonance and “negative” change, contending tears come “at the cost of the loss of the story and the fantasy.” He notes that fantasy has an element of impossibility. “The fantasy is fulfilled, but the fulfillment is precisely implausible…It can…last only as long as the fiction.”	Crying ties to revision of expectations when the story ends—“a mark of fulfillment…and its loss—the story and the fulfillment are soon both over.” Barbalet (2005, p. 128–131) concludes, while *“joyful weeping [may] register a positive transformation of self, just as tears of suffering register loss as a negative transformation of self,”* in all cases, crying *“can be regarded as essentially expressing transformation.”*
Religious experience	James (1958, p. 213) tied “the gift of tears” to religious experience, noting that they occur when one has held false beliefs or “moral stagnancies” (p. 213).	Tears arise when, through “gradual growth or by a crisis” (p. 213), one must “give up…let go (their) hold” creating *“change in terms of the old self”* culminating with final “conversion” (pp. 169–175). “To get to it, a critical point must usually be passed, a corner turned within one. Something must give way” (p. 171).

a *Division of cases based on two-factor theory of crying (Efran and Spangler, 1979). Explicit mention of self change/“transformations” in italics*.

**Figure 1:**
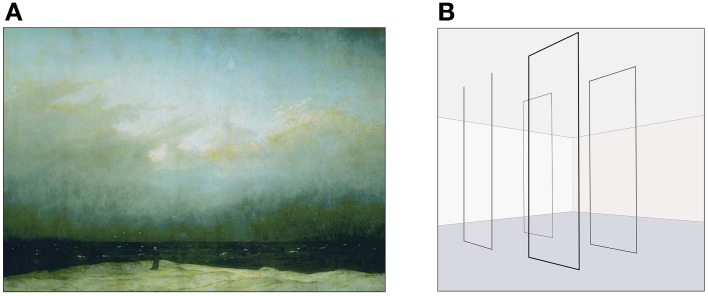
**Examples of tear-inducing art**. **(A)** Caspar David Friedrich, Der Mönch am Meer, 1808–1810. (Oil on canvas, 110 × 171.5 cm. public domain image, Wikimedia commons). **(B)** illustration depicting archetypal Fred Sandback work (image created by first author).

For example, this progression can arise from abstract painting, where according to Elkins (2001), the sheer austerity or opacity of a work may render a viewer unable to understand or process its meaning, leading to a “turning point” where one can either desist or undergo reflection and revision in one's expectations for perceiving the art—ending in the latter case with crying. Similar discussion can be found with Postmodern sculpture, where for example an account by Fraser (2006), involved a work by the American artist Fred Sandback composed only of string strung floor to ceiling, demarcating ephemeral spaces (Figure 1). In her encounter she recalled feeling that the works were so devoid of substance that they led her to question the ontological viability of art, causing a subsequent questioning of art making, and—because she was herself an artist—causing a re-evaluation of herself. The same progression can be found in reactions to the mimetic content of numerous Romantic, Renaissance, and Baroque paintings (Figure 1), which somehow pose a theme or scene that challenges the viewer. This can also be traced in discussions of music, literature, opera, as well as even in happy or moving cases (see “beauty, perfection, overwhelming stimuli,” and “happy endings” in Table 1). In every instance there is some manner of expectation, some discrepancy and some adjustment of perception, ideas, and self.

### Crying and art: indicator of insightful or “aesthetic” experience?

More important than these examples in and of themselves, is the suggestion of what they may indicate when considered within the frame of information-processing/art experience. As recently explored by Pelowski and Akiba (2011) in a review and model for art perception, it is this same outcome of self/schema change and this same cognitive chain of events—specifically as they do occur in such static contexts—that form the essential basis of numerous conceptions of insight and adjustment within perception itself.

Notably, Gadamer (1984; also Rothbaum et al., 1982) argued that this movement between discrepancy and self/schema change represents the distinction between “facile” interpretation and deepened perception or personal growth. Torrance (1979) focused on these same events in review of epiphanic or “aha” encounters in education. The same progression was highlighted in a recent cognitive/neurological review of insight by Sandkühler and Bhattacharya (2008). *And within discussion of art*, it is this overcoming of discrepancy and self/schema change that represents the distinction between superficial or abortive interaction and an “aesthetic”—i.e., novel, profound—experience (Dufrenne, 1973; Dewey, 1980; Adorno, 1984). Lasher et al. (1983; p. 197; see also Funch, 1997 for review), give an almost verbatim reprisal of the art examples above. They argue, there is “a representational conflict” between schema and perception, leading to some discrepant response. “There is next the discovery of a mental resolution which is immediately recognized as being correct in that it dissolves the preceding (difficulty),” ending in novelty or insight. Noting this similarity, Miceli and Castelfranchi (2003, p. 261; see also Barbalet, 2005) have gone so far as to explicitly suggest that the “special frustration” and final resolution of these concepts may directly correspond to, *and perhaps be indicated by*, crying—especially with art.

This then raises an intriguing potential for empirical study, which the remainder of this paper will explore. While each account of crying is unique, by taking this emergent cognitive theory and considering via parallel discussion of insight and aesthetic response, tears may provide a means of empirically dividing and evaluating art or other perceptual experience. In fact, Frey and Langseth (1985; Labott and Martin, 1988) have argued that tears, or as I consider here even “feeling like crying,” may represent *one of the only* physical responses specifically linked to self/schema change, and therefore one of the few explicit indicators of this progression. Crying may also be of specific importance to more ecologically valid museum-based study, because it is in such contexts where it is difficult to unobtrusively assess psychological response (e.g., Jacobsen, 2006). The stages posited for crying have also been argued to be key components of art evaluation, understanding and emotional arousal (Pelowski and Akiba, 2011). Therefore, tears may also offer a frame for organizing numerous contextual factors in experience. By connecting crying to parallel discussion of insight, articulated via art, this discussion may also allow new understanding of crying itself.

## Combined model of crying and “aesthetic”/insight experience

As noted above, we (Pelowski and Akiba, 2009, 2011) recently proposed a cognitive model for art viewing that can serve as a frame for considering crying's perceptual importance. This model follows a recent trend in discussion of art viewing which explores this process from an information-processing or cognitive basis (e.g., Lasher et al., 1983; Leder et al., 2004). It addresses the cognitive process of schema-/self-change argued to be crying's cognitive basis, while also making connection to insightful/transforming or “aesthetic” experience. Even more, this model incorporates the two key elements noted for crying—discrepancy and triggered reassessment—as key demarcative points for understanding experience.

**Figure 2:**
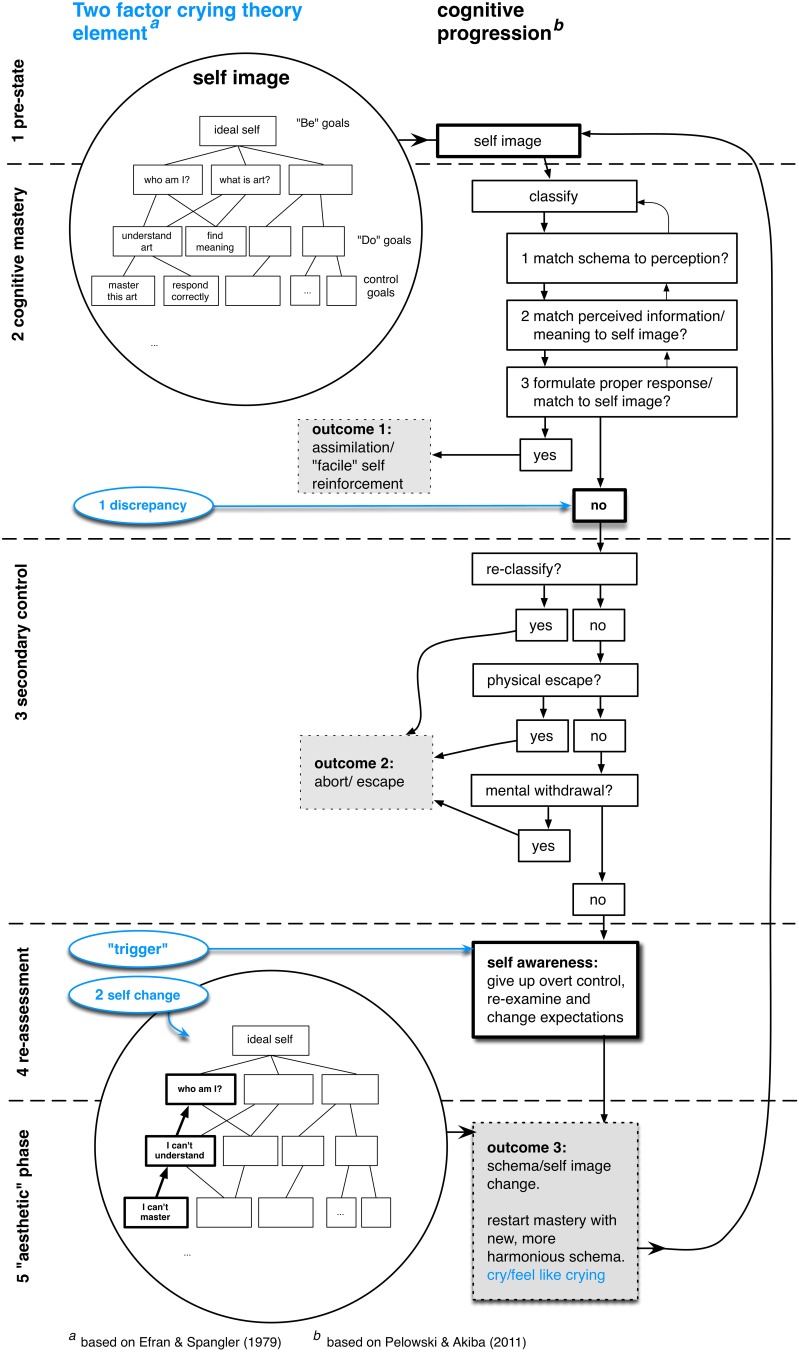
**Cognitive Flow Model of schema change and insightful/“aesthetic” experience, with major factors of crying highlighted**. The main flow model (based on Pelowski and Akiba, 2011) is depicted on the right, and can be read from top to bottom, with major stages denoted on the far left and three posited outcomes depicted in gray boxes. The “hierarchical self image” (adapted by the author from Carver, 1996), upon which the cognitive progression is expected to be based, is shown in the top- and bottom-left circular insets. Main crying elements (from the two-factor crying theory, adapted from Efran and Spangler, 1979) are shown in blue.

The model is shown in Figure 2, and can be read from top to bottom. This also incorporates the key crying factors (blue circles) with arrows connecting to the relevant model sections. As in the crying discussion, the model's essential mechanism centers on the process of forming and matching expectations or schema to a “reality” presented by a stimulus. The model however expands discussion to five stages, with three outcomes, each with specific importance for perception/art.

### Stage one: pre-expectations and self image

The model begins with explicit discussion of schema/self. As also noted for crying, before perceptual activity viewers are argued to hold a set of postulates directing behavior, conceptions or expectations regarding all life elements, as well as likely response to the outcomes of interaction. Building from the crying theory, these postulates can and should be given specific form and specifically tied into a broader conception of the self. Following Carver (1996), these postulates collectively can be said to combine to form an individual's “ideal self image” and might best be conceptualized in a hierarchical arrangement (top-left inset, Figure 2) with a collection of ideal concepts that the individual aspires to forming the core of the self, branching off into goals or schema for their pursuit and reinforcement (“do goals”) and further divided into local expectations or schema for specific behaviors and perceptions in individual tasks (“be someone connected to art” > “do understand artworks” > “find meaning in a particular painting,” etc.). In this way, all behavior, through attachment between low-level schema and the core self, entails application of some self-postulates, and forms the basis for the following stages.

### Cognitive mastery

First, upon encountering a stimulus, whether life event or work of art, individuals immediately employ this hierarchical self in the first main model stage. In a process called “cognitive mastery” by Leder et al. (2004) in their own model of art perception, individuals attempt to directly control interaction by classifying, evaluating and responding based upon pre-expectations and in such a way as to reinforce the existing schema and self. In practice, while a viewer of the movie noted above might evaluate a character, consider their life situation and future prospects and engage with the story based upon a pre-conceived notion of how such a character's life should proceed, viewers of a painting might evaluate a work for its semiotic or conceptual meaning or appraise formal elements, based upon what one expects or have been trained to perceive in art. This occurs while individuals may simultaneously observe their own reactions, or consider implications of assessed meaning, so that these too fit and reinforce the self.

However, while success at this stage may constitute an evolutionarily-derived goal of processing, and—as it leads to ease and understanding—may itself be pleasurable or rewarding, if experience does end in cognitive mastery it is also essentially an act of circularity. This requires viewers to assimilate perceptions or actions to existing schema, and thus occurs at the cost of new perceptions or concepts. The ground for this claim is connected to strategies for protecting the hierarchical self (see Pelowski and Akiba, 2011 for review). As was mentioned above, viewers use a wide range of measures to defend against threat to the self image by diminishing potential for discrepant results. Individuals are also predisposed to attend to information that fits their self model and to ignore that which does not (also Epstein, 1973; Lawler and Thye, 1999). This is often coupled with pre-attitudes—a stimulus is “not important,” one does not need to control or understand—that aid in diminishing need for engagement. In turn, experience that ends in this stage becomes a self-protectionary, “facile” interpretation—what Dewey (1980, p. 24) called “a dead spot in experience.” It is therefore at this point that both art and crying models raise importance of discrepancy. This represents cognition of something outside existing schema, and acts as a prompt to knock an individual out of overt control.

### Secondary control

When discrepancy does occur and cannot be easily assimilated, viewers enter into the period of anxiety and tension in which they fight or repress discrepancy, and which was also noted by crying models. While these models only note basic need for this period, this stage, called “secondary control” by Rothbaum et al. (1982), also marks the second possible outcome of experience, with key importance in terms of appraisal/understanding. Where before a viewer had structured a task as an exercise in self-reinforcement, this stage carries with it an implicit acceptance of insufficiency and need for protecting the self. According to Folkman et al. (1986, p. 1000), the individual, on some level, has “appraised (the) encounter as having to be accepted.” Yet acceptance, because it would involve refutation of schema, would endanger the self-image. Viewers are left with a choice between maintaining a sense of mastery in actions or conceptions or relative importance of the stimulus or context. Because the latter is not often tied to higher-level goals, individuals typically discard or diminish the environment and in so doing preserve the integrity of their own, failed, self. This typically involves a progression of strategies, also noted in crying: (1) re-classification of stimulus/context (“the doctor is wrong,” “it's not art”); (2) physical escape—leaving, fidgeting, shifting attention; and finally (3) mental withdrawal, reducing impact of a stimulus by disengaging it from one's self—“it's only art” (see also Steele et al., 1993).

Crying literature also notes self-protection. Beyond common discussion of repression of a life event that portends unwanted change, Kagan (1967) and Suls (1972) also note forced smiling and laughter in individuals who end in secondary control, and an inverse correlation to crying. Goffman (1974, p. 353) has tied these responses to self-protectionary re-classification, “guising” a situation as lacking any seriousness and therefore threat. This outcome is also often coupled with negative emotions—sadness, anger (Silvia, 2009)—as well as negative assessments (Becker, 1982), and which are often noted with difficult events or works of art (Pelowski and Akiba, 2011).

### Self-awareness and schema change

It is only in cases when individuals do not escape that they come to the last demarcative point of this progression. In these cases, individuals find themselves in the intractable position mentioned above—unable to directly resolve discrepancy through direct mastery and also unable to disengage. This position is likely directly due to relative self-importance. For individuals who find themselves in an interaction where success constitutes a core self component, the environment cannot be easily dismissed or discarded without reflecting poorly on the efficacy of one's self. This intractable position can then be resolved in one of two ways. Either conditions of the environment change allowing a positive resolution—as might be expected in cases of crying events (Miceli and Castelfranchi, 2003). Transversely, where external reprieve cannot be found one is instead left with no choice but to seek perceptual or expectation adjustment.

While crying discussion most often frames this adjustment as a point of defeat, forcing a switch from repression to acceptance, this outcome is also central to discussion of insight. Essentially what is required is a new means of addressing the discrepancy or object itself. In turn, the means of accomplishing this is through a switch in one's manner of evaluation from direct attempt at control to metacognitive appraisal. As put by Torrance (1979, p. 182), within this switch one “looks outside” an intractable problem to the entire “system” or basis for engagement, thereby allowing for substantive changes to the entire ground (i.e., the self structure) for their perceptions. This in turn requires some amount of self-awareness. Self-awareness serves as a catalyst for inducing an outcome in a discrepant encounter (Steele et al., 1993). While leading individuals who entered a situation already employing self-protectionary expectations to abort through reclassification/escape (Ingram, 1990), it induces individuals who cannot abort to undergo reflection, focusing attention on personal limitations, increasing perceived uncontrollability, and causing schema revision (Rothbaum et al., 1982).

As was also mentioned for crying, this switch is often also tied to some external “trigger” that does unavoidably consummate previous failed attempts at control. To take examples from crying literature: the death of a character, “divorce papers in the mail,” “seeing mother in hospital hooked up to machines”; or even happy change, “learned Mom's x-rays showed nothing serious” (Frey and Langseth, 1985, p. 85). However, as discussed in Pelowski and Akiba (2011), the key element for such perceptions is arguably ability to make one aware of their actions or expectations, thus forcing perception back to the self. As often occurs in context of art, self-focus can also be triggered by numerous environmental factors without change in a stimulus—a mirror, audience, the sound of one's voice; even a painting “watching you” (see also Duval and Wicklund, 1972)—all of which may function to shift control from assimilation to a metacognitive plain.

### “Aesthetic”/insightful experience

By acknowledging and reflecting on one's previous actions or conception one can then make modifications, leading to the final stage. This too may take a number of forms: from admitting impossibility of some previous expectation, to modifying one's perceptual approach (see painting in Table 1), to undertaking a higher level reassessment of concepts. In each case, via the tie of lower level schema to higher “be” and “do” goals, one can be said to alter some component of their hierarchical self structure (lower inset, Figure 2), therefore creating a “transformation” noted above. When considered from a perspective of information processing, the key importance of this adjustment is that it allows one to essentially reset engagement and re-enter the cognitive mastery stage while employing a modified set of schema. This may allow for improved/deepened interaction or for attention to previously repressed elements (Sarason et al., 1996). In this way, viewers can be said to learn from within their interaction or to arrive at novelty, epiphany or insight. The resetting, if it is particularly profound, is also tied to experience of harmony as well as catharsis, pleasure (Steele et al., 1993). Therefore, in the context of art it is this outcome that Dewey (1951) originally called the “aesthetic phase” of experience.

It is also this outcome that would coincide with crying. With discrepancy, a viewer is argued to experience sympathetic nervous system activation—increasing heart rate, electrodermal response. This further increases with movement to secondary control. In turn, the moment of schema change has been shown to coincide with both sympathetic and parasympathetic response. Gross et al. (1994, p. 466) contend, it may in fact “be precisely this coordinated activation …that (can) make (this) such a potent physiological (experience).” This is then followed by a period of parasympathetic latency, where crying literature as well notes feelings of catharsis, pleasure (Cornelius, 1997), and tears.

### Empirical importance of crying within the model of aesthetic experience

This frame then raises a number of suggestions regarding crying's empirical significance. First, as outlined in Figure 2, the model gives a format for achieving adjusted perception, involving five major stages. These are argued to occur in order, and one is required to move through the full progression in order to arrive at the aesthetic, and potentially tearful, end. It is essentially in getting to the final outcome, overcoming strategies of self-protection or bypassing secondary control, that the above-cited authors argued to be the goal of experience. This is also an important conclusion in art, which is often designed to elicit such responses (see Pelowski et al., 2014 for review).

**Figure 3:**
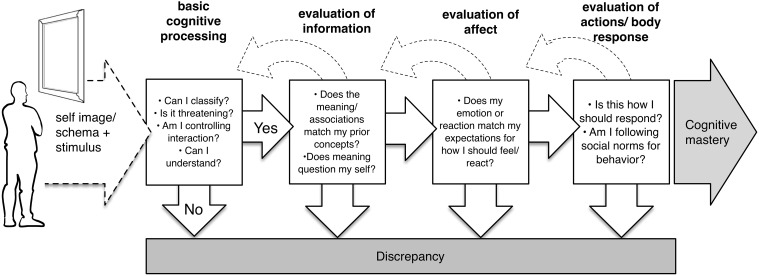
**Varieties of assessment that may lead to discrepancy within the cognitive mastery stage**.

This is not to say that schema change would *always* end in crying, or that the stages are so pronounced in all cases. The process of self-application would occur in a continuous cycle of interactions, with constant small adjustments. However, given the cognitive basis, whenever crying does occur it should coincide with some schema/self revision, and thus theoretically denote an insight experience^2^. Nor again do discrepancy/tears need to arise due to analysis of information. Ferreira et al. (2001) note that individuals perform numerous levels of processing when encountering a stimulus. Cognitive mastery involves a series of cyclic checks, with an individual evaluating in parallel the informational content, impact of information on the self, and one's behavior or response (see Figure 3 for a description of several such assessments), with any element able to induce discrepancy. Labott and Martin (1988) add that even knowing a conclusion, such as in a previously-viewed movie, does not impede crying response. Rather, to the extent that viewers allow themselves to suspend disbelief, form personally-important connections, and retrace the cognitive process, they may re-experience the same end. In addition, another main addition of the model is its explanation for two other, earlier outcomes—(1) facile control and (2) negative or discrepant self protection—which are also major outcomes in need of explanation within perception and especially art, and which may preclude crying.

**Figure 4:**
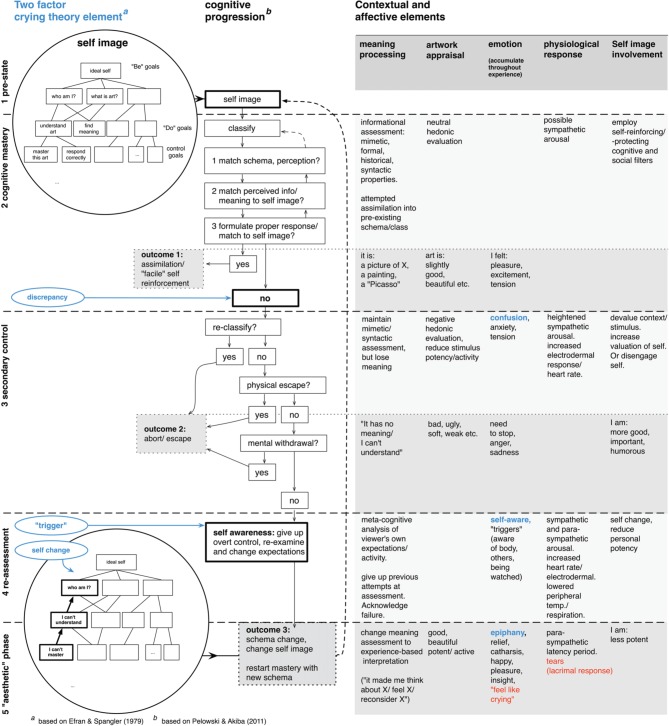
**Cognitive Flow Model of insightful/“aesthetic” experience with hypothesized factors for each stage**. Posited emotion, artwork appraisals, mode of meaning assessment, physiological reactions, and self image-related processes are shown on the right side, corresponding to the model stage at which they would be expected. Emotional factors posited to be main empirical indicators of each outcome highlighted in blue. Feeling like crying/physical tears responses shown in red.

Perhaps most important, when we do return to consideration of art or other static media, is the number of other factors that may be organized with the model and crying response. Because outcomes and their underlying stages occur in order, the model can be used to form hypotheses for a number of behavioral measures which coincide with, and partially define, each stage. These are listed in Figure 4. By exploring these factors via self-report, and by using crying as well as other main aspects in the two-factor model as points of demarcation, this may allow a frame for identifying specific outcomes of experience.

#### Emotion

First, while it is a core argument that cognitive rather than emotional processes are the cause of crying, emotion and other experiential factors, may provide one of the best measures for this progression. Specifically, the model proposes correlations between crying and: (1) anxiety, tension, confusion, stemming from initial discrepancy; followed by (2) need to escape/abort, related to secondary control. In the case of crying, this should be followed by (3) self-awareness, motive assessment, and awareness of triggers; finally concluding with (4) relief as well as epiphany or insight, marking the aesthetic end. On the other hand, experience that does not end in crying would be expected to show a halt in this progression at either cognitive mastery—with lack of confusion, anxiety, and later factors—or at aborted secondary control—with initial confusion/anxiety but no epiphany or self-awareness. These additional key factors (i.e., confusion, self awareness, and epiphany), marking proposed major points of transition within the model are also highlighted in blue in the right column of Figure 4 (see also Pelowski et al., 2014). This contention is also supported by previous literature on crying, which does show that, following discrepancy, inability to revise expectations and complete schema change is notable for its inverse correlation with tears (Dombeck et al., 1996). Literature also suggests that viewers who eventually cry may show greater sympathetic response in initial evaluation (Gross et al., 1994; Rottenberg et al., 2002), suggesting discrepancy within this stage.

#### Appraisal

The same main divisions should also be found in art/object appraisal. Experience that ends in mastery should show either a rather neutral evaluation or negative devaluation, diminishing—as self-protection—its potential threat. This might also arise in pre-expectations, with viewers in the facile category holding attitudes—art is not important, and has no connection to their life—diminishing need to actively engage. Given discrepancy, self-protection is expected to become even more pronounced, and experience ending in secondary control should show correlation to negative evaluation. This may also manifest in devaluation of the “potency” and “activity” of a stimulus, as evaluated via the Semantic Differential technique (Osgood et al., 1957). This approach, in which individuals use a number of adjectival pairs (e.g., good–bad) for assessments, has been shown to be an effective means of considering nuanced evaluation (Leder et al., 2005; Silvia, 2005). Even more, adjectival scales are routinely shown to coalesce along three factors—regarding relative potency, activity and hedonic quality (evaluation). In turn, measures—e.g., “weak,” “superficial”—that routinely refer to potency/activity areas have been argued to suggest amount of adjustment made or that must be made to a stimulus and therefore relative importance/danger to the self (Carroll, 1959). Berlyne (1974), in the context of art, also notes that negative evaluation with potency factors suggests unresolved “uncertainty” between perception and expectations, which might especially surface in secondary control. Becker (1982) also argues that secondary control should tie to devaluation of hedonic aspects—beauty, goodness—again reflecting protection of the self. Crying, on the other hand, may coincide with positive hedonic, potency and activity assessment, reflecting acknowledged ability of a stimulus to profoundly affect one's self.

#### Meaning/understanding

Outcomes should also involve three distinct approaches to assessed meaning: (1) initial evaluation in cognitive mastery involving, in the case of art, direct appraisals of formal elements—mimetic, semiotic, historical; followed by (2) a period of meaninglessness in secondary control; and (3) concluding with a new interpretation involving a fundamental shift from form/information to assessment involving self-reference. In the arts, this change may often take on a specifically important character. Forced from direct informational analysis, viewers may adopt what Dewey (1980) has called an “experiential” approach, whereby the “meaning” of a work comes to be equated to this entire process of change and personal impact or stimulus-induced insight or learning (see for example, Fraser, 2006 above). Within discussion of art, such progression from direct or unsuccessful interpretations to an enjoyment based on the act of looking or impact on the self has been given as one of the main goals of art education/experience (Pelowski and Akiba, 2011). In crying discussion as well, Martin and Labott (1991) have shown correlation between tears and deepened recall of individual experience, suggesting such connection.

#### Schema/self change

We also have proposed specific evidence for self transformation. Literature suggests that self change might again be meaningfully recorded by use of Semantic Differential, in this case regarding pre- and post-experience comparison in assessments of the term “yourself.” Miceli and Castelfranchi (2003; see also Bem, 1967) argue that the result of a self/schema transformation, occurring as a viewer acknowledges discrepancy and gives up control, might be specifically reflected in a reduction of potency in this evaluation.

#### “Feeling like crying” vs. physical tears

Finally, the above distinctions may be indicated by simply “feeling like crying.” Studies (Williams, 1982; Ross and Mirowsky, 1984; Vingerhoets et al., 1997) suggest that individuals, especially males, often arrive at the point of feeling like crying yet repress actual tears, presumably because of social stigma, and to the extent that repression becomes so established that this can occur without conscious effort. It is therefore feeling like crying, rather than overt weeping, that is argued to mark culmination at schema change (Efran and Spangler, 1979; Labott and Martin, 1988). Studies comparing overt tears and “feeling like” crying (Lombardo et al., 1983; Kraemer and Hastrup, 1986) or different intensities—“weeping” vs. “moist eyes” (Williams and Morris, 1996)—as well show no differences in elicited emotion or cognitive elements.

## Materials and methods: study 1

To conclude this paper, the above model hypotheses were applied in three exploratory studies within museums, beginning with perhaps the most notably tear-inducing contemporary artwork.

**Figure 5:**
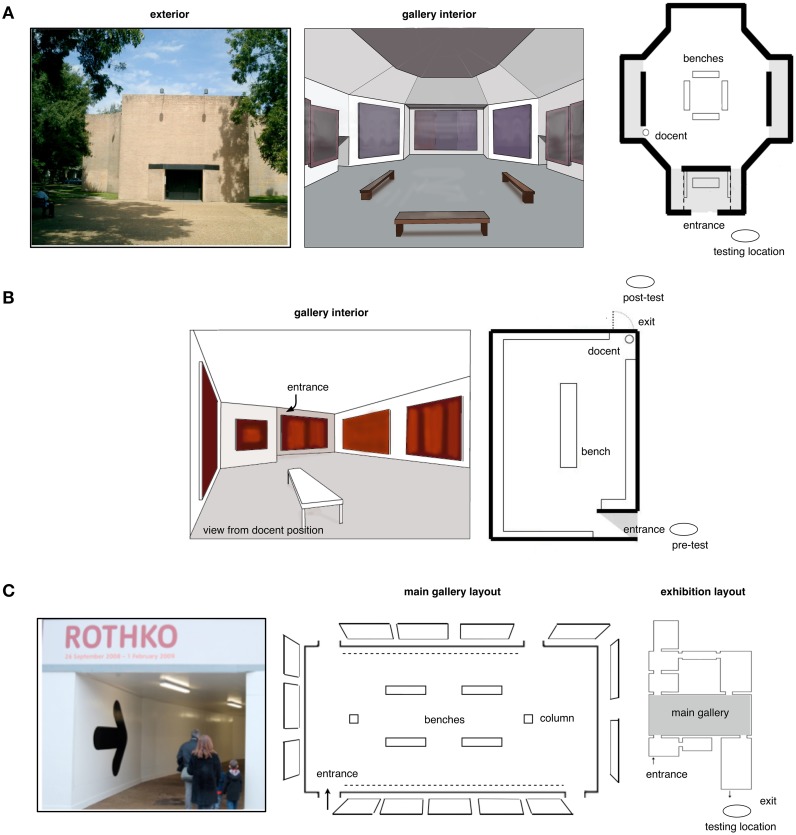
**Study locations**. **(A)** the Rothko Chapel, Houston Texas. **(B)** Rothko Room, Kawamura DIC Prefectural Museum of Art, Japan (prior to 2007 remodeling). **(C)** Floor plan for “mark Rothko, the late paintings,” Sep. 2008—Jan. 2009. Tate Modern, London. (All photos/illustrations by first author).

The artist Mark Rothko is an important representative of the American abstract expressionist movement of the 1960's. His paintings are often noted for their beauty, and are a particular draw among many viewers in museums of Modern art (Elderfield, 2005). What makes him compelling here, however, is anecdotal relation to crying. While Rothko is a fixture of Modern Art museums, one particular gallery—an octagonal room holding 14 of his paintings in Houston Texas—the so-called “Rothko Chapel” has also taken on a particular notoriety for tear elicitation. According to Elkins (2001, p. 4), “it is likely that the majority of people who have wept” over 20th century art have done so in front of these paintings. Even more interesting is the fact that the reasons for these reactions are not immediately clear. The artworks, ~2 × 3 m purple/black color fields (Figure 5), although representative of Rothko's mature style, are also quite simple. While conceived as “painting” in the classical sense, they do not portray any particular mimetic depiction, and critics agree that the paintings often lose any semblance of such mimetic meaning, eventually coming to be viewed not as pictures but as “just” paint (Jones, 2002). This reaction, however, when connected to our model, may be the driving force for the experience.

**Figure 6:**
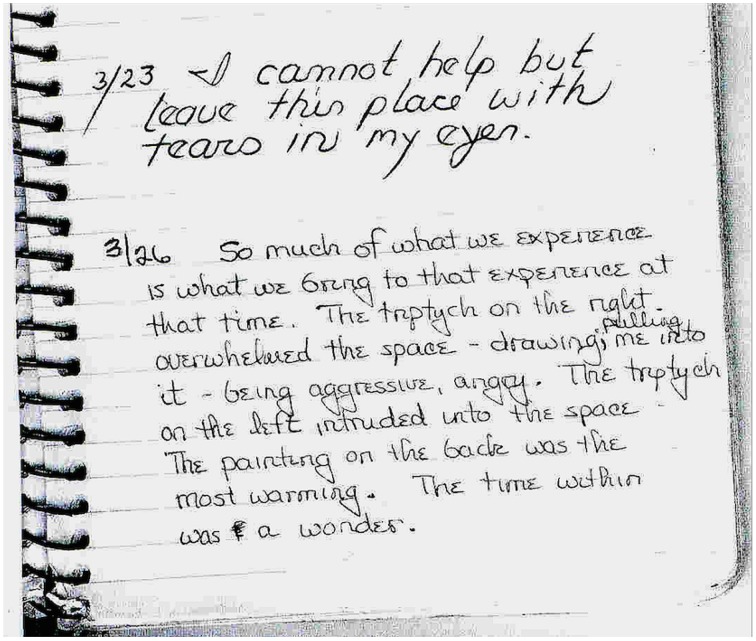
**Comments in the visitor's log book noting crying and self-referential or experience-based interpretation of artwork meaning, Rothko Chapel, Houston Texas**. (photo provided by curator to first author, 2006).

As described by several writers (Nodelman, 1997; Elkins, 2001; Elderfield, 2005), upon entering the room, due to the paintings' monumental quality and minute brushstrokes, viewers often have a sense of profound significance. As viewers attempt to fit the paintings together and understand their collective meaning, the paintings' redundancy and opacity can reject mimetic interpretation, leading to a “turning point” between escape or reconsideration. In the latter case, experience then comes to parallel the above discussion. Eventually, notes Elderfield (p. 112), “we are reminded that we are alone in front of the paintings.” “Frustrated and thrown back to yourself,” Nodelman (p. 330) adds, viewers “become the center of the room…. (The art) forces disturbing questions about the nature of the self and its relation to the world.” And this induces an interpretive change in the viewer, in which “the experiential structure of the installation (itself) is transformed” (p. 342). As a comment in the visitors' log concludes (in (Barnes, 1989); see also Figure 6), summing up this paper's discussion, ″being in the room is an emotional experience in which you either face your innermost self”—and potentially feel like crying—“or leave in incomprehension.”

The room itself was also especially suited to controlled empirical study. It contained only a bench and the paintings with no extraneous information—labels, audio guide, windows, artworks—essentially duplicating a laboratory setting. The abstract paintings, because of their absence of mimetic content, were also unlikely to evoke highly personal associations that might cloud between-subject analyses. This allowed better targeting of the posited underlying cognitive processes (Jacobsen et al., 2006). As noted above, the museum setting was also an important element. As opposed to movies or other temporal media that might use a story to evoke crying response, in the case of static art, the laboratory—with digitally reproduced paintings—is not likely to evoke such a personal, powerful reaction, necessitating focus on a more natural, spontaneous experience (Jacobsen, 2006).

### Participants

To investigate interaction with the art, 21 individuals (*Mean* age = 48; 11 females) were recruited on site, constituting all patrons who consented to participate from among all those who visited within the testing period (~60% acceptance). Selection was also confined to only single or paired adults, following Packer and Ballantyne (2002) who suggest that it is only these individuals who are likely to engage to a sufficient depth to routinely allow for the targeted experience.

### Procedure

Procedure followed a method previously applied with success in museum studies (Pelowski et al., 2012, 2014), designed to allow the capturing of a “natural” experience without intrusion from researcher or apparatus. Participants were approached by a researcher in the courtyard before entering the building, which contained only the gallery and foyer. The researcher explained that they were conducting research on the experiences had with the art, and asked guests if they would be interested in answering a survey following their viewing. Those who consented were given a pre-test, targeting general art expectations (requiring about 10 min, see *Measures* below). Participants were then asked to enter the gallery and view the art in whatever manner and for whatever duration that they desired. Once finished, participants returned to the testing location where they completed a post-test (~20 min) and a brief exit interview. Participants were timed, however they were not observed, inside the gallery. Access was free and visitors were not compensated for participation. This study, and the following Studies 2 and 3, were approved by the ethics committee of Nagoya University and by the participating museums.

### Measures

The measures for the study were designed to allow investigation of two main questions: (1) was it possible to record evidence for feeling like crying when viewing the art, and how would individuals respond to this question? (2) would participant answers regarding the crying and other experiential/evaluative factors show patterns of responses suggesting a full progression through the hypothesized model stages, and which might suggest correlation between “aesthetic” and crying experience? Further, could a demarcation be made between the crying outcome and other “facile” or “abortive” outcomes when viewing the art? Because this study was largely exploratory, the above questions were assessed via questionnaires containing a large number of measures, targeting the multiple factors posited above. These are discussed below, with measures listed in Tables 2 and 3. This also meant that the study was intended from the onset as exploratory, with statistical examination used largely as a guide for a qualitative assessment of the interaction with this specific art.

**Table 2:** **Correlation of reported “felt like crying” and other experiential/emotional factors in three encounters with museum art**.

		**Rothko chapel, USA (*N* = 21)**		**Tate modern, England (*N* = 28)**		**Kawamura DIC, Japan (*N* = 30)**	
**τB**	**95% CI^b^**	**τB**	**95% CI**	**τB**	**95% CI**
**COGNITIVE MASTERY/DISCREPANCY^a^**						
Anxiety	0.252	[−0.16, 0.59]	0.159	[−0.17, 0.46]	−0.179	[−0.44, 0.11]
Confusion	0.239	[−0.13, 0.55]	0.066	[−0.26, 0.37]	−0.204	[−0.47, 0.09]
Tension	0.506^***^	[0.23, 0.71]	0.073	[−0.27, 0.40]	------	------
Surprise	−0.131	[−0.48,0.25]	------	------	------	------
**SECONDARY CONTROL**						
Need to leave	0.604^***^	[0.41,0.75]	0.299	[0.04,0.52]	−0.025	[−0.33, 0.29]
**SELF-AWARE REFLECTION**						
Self-awareness	0.660^***^	[0.53, 0.76]	0.330^**^	[0.09, 0.53]	0.410^***^	[0.15, 0.62]
Felt being watched	0.088	[−0.27, 0.43]	0.293	[0.04, 0.51]	0.322^**^	[0.01, 0.58]
Changed my mind	0.000	[−0.36, 0.36]	0.099	[−0.22, 0.40]	------	------
Examined motives for viewing	0.065	[−0.32, 0.44]	−0.076	[−0.40,0.26]	------	------
Felt the paintings watching me	0.091	[−0.31, 0.46]	------	------	------	------
Aware of my actions	0.166	[−0.17, 0.47]	------	------	------	------
**AESTHETIC PHASE/INSIGHT**						
Epiphany	0.469^***^	[0.22, 0.66]	0.529^***^	[0.32, 0.69]	0.339^**^	[0.05, 0.58]
Understood artist intention	0.212	[−0.16, 0.53]	0.394^**^	[0.17, 0.58]	0.282^*^	[0.01, 0.51]
Sadness	0.421^**^	[0.14, 0.64]	0.234	[−0.08, 0.51]	0.233	[−0.06, 0.49]
Happiness	0.382^**^	[0.13, 0.59]	0.243	[−0.01, 0.47]	0.224	[−0.02, 0.45]
Relief	0.427^**^	[0.20, 0.61]	0.197	[−0.14, 0.49]	------	------
Time	0.231	[−0.06, 0.48]			0.437^**^	[0.18, 0.64]

Ratings made on 9-point scale (0 = “no such feeling,” 8 = “the most intense such feeling in my life.”).

* (Bold) Correlation significant at p < 0.05 (2-tailed), non-parametric Kendall tau-b.

** p < 0.01;

*** *p < 0.001*.

a *Divisions based on proposed stages of aesthetic experience model (Pelowski and Akiba, 2011)*.

b *Confidence Intervals calculations based on Asymptotic Standard Error*.

**Table 3:** **Correlation of “feeling like crying” with artwork evaluation in three encounters with museum art**.

	**Rothko chapel, USA**		**Kawamura, Japan**		**Tate modern, London**	
	**(*N* = 21)**		**(*N* = 30)**		**(*N* = 28)**	
	**τB**	**95% CI^c^**	**τB**	**95% CI**	**τB**	**95% CI**
**EVALUATIVE SCALES**^a^						
Good: bad	−0.325‡^b^	[−0.51, 0.10]	−0.441^***^	[−0.61, −0.23]	−0.423^***^	[−0.58, 0.24]
Beautiful: ugly	−0.365‡	[−0.65, 0.00]	−0.531^***^	[−0.66, −0.37]	−0.456^***^	[−0.60, −0.28]
Meaningful: meaningless	−0.382^*^	[−0.62, −0.07]	−0.394^**^	[−0.59, −0.15]	−0.613^***^	[−0.71, −0.49]
Happy: sad	−0.067	[−0.36, 0.24]	------	------	------	------
**POTENCY SCALES**^a^						
Intimate: remote	−0.385^*^	[−0.62, −0.09]	−0.384^**^	[−0.60, −0.12]	−0.18	[−0.46, 0.14]
Sincere: insincere	−0.558^**^	[−0.69, −0.39]	−0.177	[−0.40, 0.07]	−0.374^**^	[−0.37, 0.24]
Deep: shallow	−0.062	[−0.45, 0.35]	−0.251	[−0.49, 0.02]	−0.307^*^	[−0.56, −0.15]
Vague: precise	−0.519^**^	[0.31, 0.68]	−0.017	[−0.24, 0.27]	−0.075	[0.04, 0.52]
Controlled: accidental	−0.167	[−0.19, 0.48]	−0.198	[−0.06, 0.43]	−0.266	[−0.51, 0.01]
Simple: complex	−0.298	[−0.01, 0.55]	−0.226	[−0.06, 0.48]	−0.053	[−0.36, 0.26]
Strong: weak	−0.234	[−0.53, 0.11]	−0.177	[−0.43, 0.10]	------	------
Unique: commonplace	−0.444^*^	[−0.64, −0.19]	−0.288^*^	[−0.49, −0.06]	------	------
Serious: humorous	−0.394^*^	[−0.64, −0.07]	------	------	------	------
Superficial: profound	−0.409^*^	[0.11, 0.64]	------	------	------	------
Clear: hazy	−0.632^**^	[−0.73, −0.51]	------	------	------	------
Full: empty	−0.299	[−0.55, 0.00]	------	------	------	------
Hard: soft	−0.199	[−0.16, 0.51]	------	------	------	------
**ACTIVITY SCALES**^a^						
Lively: calm	−0.448^*^	[−0.66, −0.18]	−0.132	[−0.39, 0.15]	−0.298^*^	[−0.53, −0.04]
Fast: slow	−0.036	[−0.41, 0.35]	−0.037	[−0.33, 0.26]	------	------
Relaxed: tense	−0.191	[−0.15, 0.49]	−0.175	[−0.43, 0.11]	------	------
Noisy: quiet	−0.195	[−0.24, 0.56]	------	------	------	------
Hot: cold	−0.21	[−0.51, 0.13]	------	------	------	------

Ratings made on 7-point bipolar scale (1 = “very good,” 2 = “quite good,” 3 = “slightly good,” 4 = “neither good nor bad”).

* (Bold) Correlation significant at p < 0.05 (2-tailed) non-parametric Kendall tau-b.

** p < 0.01;

*** *p < 0.001; ^‡^p < 0.1*.

a *Division of scales into Evaluative, Activity, and Potency based on Osgood et al. (1957)*.

b *Negative correlation coincides with leftward term on scale, positive correlation coincides with rightward term*.

c *Confidence intervals calculations based on Asymptotic Standard Error*.

#### Pre-expectations

The pre-viewing questionnaire consisted of a series of 5-point scales (“1” = strongly disagree, “2” = disagree, “3” = neither agree nor disagree) addressing expectations for viewing, art understanding/comfort and relation between art and self, based on those reviewed in Pelowski and Akiba (2011).

#### Artwork appraisal, self appraisal

The pre-test also asked viewers to evaluate the term “yourself” via 22 adjectival pairs (listed in Table 3) separated by 7-point bipolar scale (1 = “very good,” 2 = “quite good,” 3 = “slightly good,” 4 = “neither good nor bad”). Upon viewing the artworks, viewers were then asked to again evaluate themselves, as well as “the artwork,” in the post-test using the same pairs. These scales were based on the Semantic Differential instrument of Osgood et al. (1957). As reviewed in above, this technique, in addition to measuring the connotative meaning of objects or concepts, was selected because of its applicability for assessing relative level of self-protection employed in interacting with a stimulus. When considering changes in self-evaluation, the technique is also argued to have specific efficacy as an indicator of transformation/schema-change, and also has widespread cross-cultural use especially with art (e.g., Tanaka et al., 1963; Berlyne, 1974). The adjective pairs were selected from the original lists of Osgood et al. (1957), with the goal of providing a representative range of scales with high loadings among the main Evaluative, Potency and Activity regions.

#### Emotional/experiential factors

The post-test also included unipolar 9-point scales (listed in Table 2) eliciting self-report on emotional and experiential factors mentioned as potential components in aesthetic/tearful experience [e.g., “while I was inside the room, I experienced (factor)”]. In this case, an answer of “0” corresponded to “no such feeling,” answers from “1”–“8” corresponded to some incidence as well as relative magnitude, with “8” signifying “the most intense such feeling in my life” (following Ekman et al., 1980; Carstensen et al., 2000).

#### Feeling like crying

The above section also included the term “I cried or felt like crying.” This was chosen, rather than attempt to monitor actual shedding of tears in the gallery, as our crying measure because of the desire to maintain a natural viewing experience. In addition, as discussed above, this approach was chosen because of ability of individuals to arrive at schema change/crying but repress physical tears. Pairing of self-report with 9-point scale has also previously been employed by multiple studies (Kraemer and Hastrup, 1986; Martin and Labott, 1991; Williams and Morris, 1996), which have found no incidence of under- or mis-reporting when compared to physiological measures.

Order of survey scales, as well as survey sections, was randomized between participants. An alpha of 0.05 was set for all results.

## Results: study 1

The art experience took on average 15.5 min (*SD* = 8.3). No significant interactions were found between demographic criteria and other measures. In addition, no subjects expressed knowledge of the anecdotal tie between the art and crying or any other above-noted reactions. While most had heard of the artist, 66% were first time visitors. No significant ties were found between foreknowledge or number of previous visits and answer to the survey questions. At the same time, 43% of subjects reported some magnitude of feeling like crying when viewing the art (*M* = 4.1, *SD* = 2.2). This response was then used as the focus for subsequent analysis.

First, correlation analyses were conducted in order to assess the basic relation of feeling like crying (hereafter, “FLC”) with other emotion and evaluation factors. Due to the ordinal nature of the scales, non-parametric analyses were used for this and all of the following procedures. Following current best practice in exploratory research (see Rothman, 1990), these analyses do not include correction for multiple assessments, but include both effect sizes and confidence intervals. The correlation analyses used Kendall tau-b, which is argued to provide a more accurate and conservative test than similar Spearman rank-order (Kendall and Gibbons, 1990). Results are shown in Table 2. Significant positive correlation was found between FLC and *tension, need to escape, change in mood*, as well as *self-awareness, epiphany, relief*, and both *happiness* and *sadness*. In artwork appraisal (Table 3), positive correlation was found between FLC and finding the art more *meaningful*, as well as with evaluating the art as more *precise, sincere, lively, clear, intimate, unique, serious*, and *profound*. Although not significant, there was also a trend between FLC and finding the art more *beautiful* and *good* (*p* < 0.10).

In order to begin assessing clustering of the above factors, viewers were then separated based on two elements—*confusion* and *feeling like crying*—argued to demarcate the major outcomes of processing experience. This led to three main divisions: (1) those who reported neither confusion nor feeling like crying (hereafter “no-confusion, no-FLC”), argued to correspond to the outcome of initial “cognitive mastery” (*n* = 6); (2) “confusion, no-FLC,” reflecting a conclusion in aborted “secondary control” (*n* = 6); and (3) those with confusion who did feel like crying (“confusion, FLC,” *n* = 8), potentially reflecting the insightful/aesthetic outcome. Again, it was posited that FLC without confusion would be unlikely (here *n* = 1). Confusion was chosen because it was expected to best indicate a discrepancy in cognitive assessment, although anxiety returned similar findings.

While these divisions result in quite small groupings, and thus extra care should be taken if making inferences, this did produce some noteworthy results. Findings show an almost even distribution into the three groups argued for in this paper. In turn, reporting feeling like crying was concomitant with discrepancy or confusion. Only one individual reporting FLC without confusion. This individual's responses largely resembled those of non-criers. The “confusion, no-FLC” and “confusion, FLC” subgroups also showed similarly high magnitudes of this feeling. At the same time, Kruskal-Wallis comparison of mean ranks between the three main subgroups indicated several significant differences. As shown in Table 4, differences were found for reported *self-awareness*, with post hoc Mann-Whitney comparison between subgroups showing significant difference between the latter “FLC, confusion” outcome and both other no-FLC groups. Significant differences were also found for *feeling the paintings watching me*, argued to be a potential trigger, and showing lowest total for the “no-confusion, no-FLC” group and higher for “confusion, no-FLC” and “confusion, FLC” outcomes. *Post-hoc* comparison for “no-confusion” vs. both “confusion” groups was also significant. Similar results were also found for *happiness* and *epiphany*, with posthoc comparison in the latter factor showing significant differences between the “no-confusion” and the “FLC” outcomes. Nonparametric Levene's test of variance of homogeneity for all factors showed no violations.

**Table 4:** **Comparison of key reported experiential factors between three hypothesized “feeling like crying/no- feeling like crying” + “confusion/no-confusion” subgroups**.

	**Rothko chapel, USA**		**Kawamura, Japan**		**Tate modern, England**	
	**Median^a^**	***H* (η^2^)^b^**	**Median**	***H* (η^2^)**	**Median**	***H* (η^2^)**
**CONFUSION**						
No confusion + no cry	0.0 (*n* = 6)	------	0.0 (*n* = 3)^c^	------	0.0 (*n* = 9)	------
Confusion + no cry	2.5 (*n* = 6)		5.0 (*n* = 16)		3.0 (*n* = 9)	
Confusion + cry	2.0 (*n* = 8)		4.0 (*n* = 9)		2.0 (*n* = 9)	
**ANXIETY**						
No confusion + no cry	0.0	5.44 (0.27)	0.0^a^^b^	6.20^*^ **(0.23)**	0.0	4.78 (0.18)
Confusion + no cry	0.5		4.0^a^		0.0	
Confusion + cry	1.5		2.0^b^		0.0	
**SELF-AWARENESS**						
No confusion + no cry	0.0^a^	10.45^**^ **(0.55)**	0.0	3.03 (0.11)	3.0	8.86^**^ **(0.34)**
Confusion + no cry	1.0^b^		1.0		0.0^a^	
Confusion + cry	6.0^a^^b^		4.0		6.0^a^	
**Trigger**						
	**“Painting watching me”**		**“Aware of others/ being watched”**		**“Aware of others/being watched”**	
No confusion + no cry	1.0^a^^b^	7.54^*^ **(0.40)**	0.0^a^	9.03^**^ **(0.33)**	0.0	6.48^*^ **(0.25)**
Confusion + no cry	2.5^a^		2.0^b^		0.0	
Confusion + cry	2.5^b^		5.0^a^^b^		0.0	
**EPIPHANY**						
No confusion + no cry	0.0^a^	9.86^**^ **(0.52)**	0.0	5.66^*^ **(0.21)**	0.0^a^	13.72^***^ **(0.53)**
Confusion + no cry	3.0		0.0_a_		3.0^b^	
Confusion + cry	5.0^a^		4.4_a_		6.0^a^^b^	
**HAPPINESS**						
No confusion + no cry	0.0^a^^b^	10.68^**^ **(0.56)**	5.0	2.5 (0.09)	2.0^a^^b^	7.06^*^ **(0.27)**
Confusion + no cry	3.5^a^		2.0		6.0^b^	
Confusion + cry	5.0^b^		4.0		5.0^a^	

*All judgments made on 9-point scale (0 = “no such feeling,” 8 = “the highest such feeling in my life”)*.

* (Bold) p < 0.05, nonparametric Kruskall-Wallis test for comparisons of mean ranks;

** p < 0.01;

*** *p < 0.001*.

a *Medians with the same subscripts differ significantly at p < 0.05, post hoc Mann-Whitney comparison, uncorrected*.

b *Effect size calculated following (Green and Salkind, 2005)*.

c *This study dropped the first outcome (No confusion + no cry) from statistical analysis within the paper discussion due to small sample size. It is shown here only for comparison purposes. Please refer to the text for statistical results from comparison between the two remaining subgroups*.

Because of the observed main division between those reporting/not reporting FLC, and in order to provide more robust group sizes, simple comparison was also made between FLC/non-FLC outcomes (Table 5). This also revealed significance regarding the same factors as above. Those reporting FLC also had significantly higher *need for escape, self-awareness* and *epiphany*. Those reporting FLC also rated the art as significantly more “good.” Although not significant, those reporting FLC also spent generally more time in the gallery (*Mdn* = 20 min) vs. no-FLC group (*Mdn* = 12, *p* = 0.17).

**Table 5:** **Comparison of reported experiential factors and artwork evaluations between viewers who do or do not “feel like crying” in three encounters with museum art**.

	**Rothko chapel, USA**			**Kawamura, Japan**			**Tate modern, England**		
	**Median**	**CI^c^**	***U*(*r*)**	**Median**	**CI**	***U*(*r*)**	**Median**	**CI**	***U*(*r*)**
**NEED TO LEAVE^a^**									
No cry	0.0(*n* = 12)	[0, 2]	18.0^**^ (0.70)	0.0(*n* = 19)	[0, 4]	105.0 (0.01)	0.0 (*n* = 18)	[0, 0]	63.0^*^ (0.46)
Cry	1.0(*n* = 9)			1.0(*n* = 11)			0.0 (*n* = 10)		
**SELF-AWARENESS^a^**									
No cry	0.0	[2, 6]	9.5^***^ (0.71)	0.0	[0, 3]	54.0^*^ (0.41)	2.0	[1, 4]	39.0^**^ (0.47)
Cry	6.0			4.0			5.5		
**AWARE OF OTHERS/BEING WATCHED^a^**									
No cry	2.0	[0, 2]	48.0 (0.10)	2.0	[0, 5]	55.0^*^ (0.40)	0.0	[0, 0]	63.0^*^ (0.46)
Cry	2.0			4.0			0.0		
**EPIPHANY^a^**									
No cry	0.5	[3, 5]	21.5^**^ (0.51)	0.0	[0, 4]	52.5^*^ (0.43)	3.0	[1, 5]	25.0^***^ (0.60)
Cry	5.0			4.0			6.0		
**ARTWORK WAS MEANINGFUL^b^**									
No cry	0.5	[0, 2]	58.0‡ (0.39)	0.0	[0, 2]	150.0^*^ (0.37)	0.0	[1, 3]	160.0^**^ (0.65)
Cry	2.0			2.0			2.0		
**ARTWORK WAS GOOD^b^**									
No cry	0.0	[0, 3]	62.0^*^ (0.44)	0.0	[0, 2]	161.5^**^ (0.47)	1.0	[0, 2]	146.0^**^ (0.53)
Cry	2.0			1.0			3.0		
**ARTWORK WAS BEAUTIFUL^b^**									
No cry	0.0	[0, 3]	59.0^‡^ (0.39)	0.0	[0, 2]	171.0^**^ (0.54)	1.0	[0, 2]	144.5^**^ (0.52)
Cry	2.5			1.0			3.0		

* (Bold) p < 0.05, Mann-Whitney two-sample rank-sum test;

** p < 0.01;

*** p < 0.001;

‡ *p < 0.1*.

a *Reported emotion/experiential factors based on 9-point scale (0 = “no such feeling,” 8 = “the highest such feeling in my life”)*.

b Artwork evaluations based on 7-point bipolar scale (e.g., 3 = “very good,” 2 = “quite good,” 1 = “slightly good,” 0 = “neither good/bad”).

c *Confidence Interval based on Hodges-Lehman estimate of median difference between populations*.

Finally, Wilcoxon matched-pairs signed-rank test was conducted on the self evaluations, looking individually at both the FLC or no-FLC reporting groups. Among FLC viewers, results did not reach significance. This group however did show a notable trend of re-evaluating their selves as more *cold* (*Z* = −1.73, *p* = 0.08, Hodges–Lehmann estimate for *Mdn* score change = 0.5 [0, 1]) as well as less *unique* (*Z* = −1.73, *p* = 0.08, *Mdn* change = 0.5 [0, 1]) following the experience. Those who did not feel like crying did show significant re-evaluations of themselves as more *humorous* (*Z* = −2.12, *p* = 0.03; *Mdn* change = 0.5 [0, 2]). One significant correlation was also found between FLC and pre-expectations, regarding the statement “I usually understand art” (τ_B_ = 0.42 [0.17,0.62], *p* = 0.02).

## Discussion

Results appear to support most hypotheses made for feeling like crying as an important phenomena within, and an indicator of, “aesthetic” experience. There was a notable percentage of viewers (43%) who responded affirmatively when asked about feeling like crying as a component of their experience. This in itself was somewhat surprising given the novelty of this question in art/perception research.

**Figure 7:**
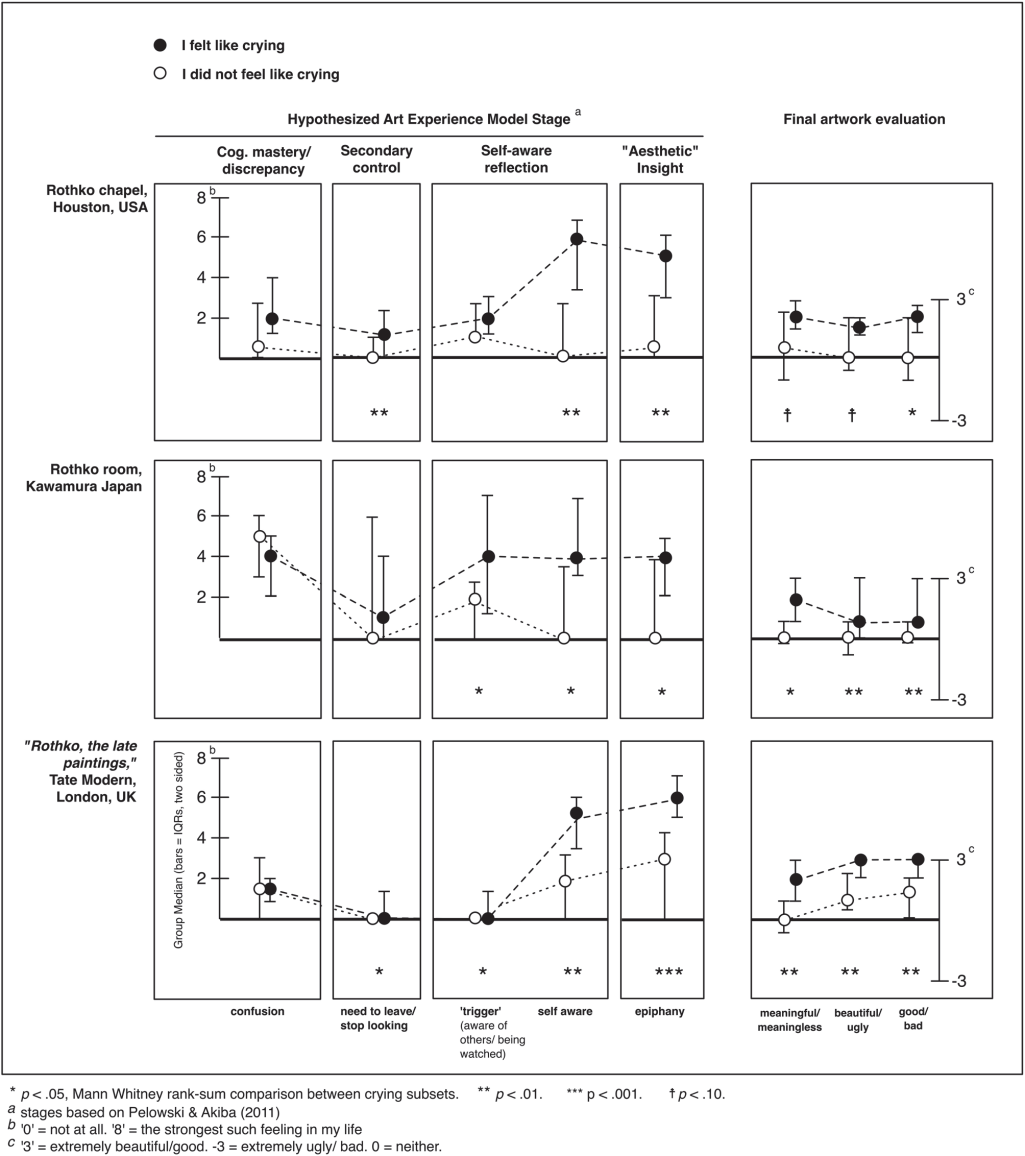
**Comparison of key emotions and artwork evaluations between viewers who did or did not “feel like crying” in three encounters with museum art**.

Even more, results showed a pattern of correlations between FLC and other factors that can be interpreted to represent the full range of stages posited in the above model. FLC showed significant correlations with: (1) *tension*, posited to occur in the initial “cognitive mastery” stage involving arousal of discrepancy; (2) *need to escape*, posited to accompany subsequent attempt at secondary control; (3) *self-awareness* suggesting switch to metacognitive or self-referential assessment; and ultimately (4) *relief*, *happiness*, and *epiphany*, argued to suggest the final “aesthetic” phase of the model. The above progression was further highlighted after dividing viewers into combinations of reported confusion and FLC. Again keeping in mind the minimal group sizes, FLC viewers did appear to show all of the elements above at significantly higher levels. On the other hand, without FLC, individuals may or may not have shown discrepancy (denoted by confusion), but did not show self awareness, happiness, relief or epiphany, and also largely did not find meaning in the art. The progression was even more clearly shown in simple comparison between FLC/no-FLC subgroups (Figure 7).

In art evaluation, FLC also showed significant correlation with a number of assessments—precise, clear, lively, serious, sincere, profound. While I make no attempt to parse these individual assessments (such an analysis should be done in future studies employing factor analysis), these responses are commonly connected to the Potency or Activity regions (Osgood et al., 1957). Following Carroll (1959), these measures can also be interpreted to suggest a greater sense of seriousness/stimuli importance and therefore may suggest potential for self-adjustment. Transversely, when considered from the direction of those who reported low incidence of feeling like crying, and therefore found the art more remote, vague, hazy, and insincere, these responses can be interpreted to reflect a lack of potency/seriousness and therefore absolution from involvement. Results also showed connections between FLC and finding the artworks more meaningful and good. These hedonic scales were also hypothesized to reflect the division between aesthetic or facile/abortive experience.

Finally, regarding the “self transformation” argued to accompany interpretive change, although results did not show significant changes among viewers reporting feeling like crying, the noted trend of FLC viewers re-evaluating themselves as more “cold” and less “unique” does provide intriguing suggestion. These terms typically have ties to relative impotency and inactivity (Osgood et al., 1957), and therefore would coincide with the hypothesis that schema change should be reflected in devaluation of these qualities. At the same time, the significant change discovered among those with no FLC is also intriguing. These individuals became more “humorous.” This term had been argued (Goffman, 1974) to represent a general attempt at re-classification of an encounter by reframing one's self in a manner that diminishes the seriousness or importance of one's involvement, and therefore reducing potential for impact on the self, as well as previously showing inverse correlation with crying (Labott and Martin, 1988). The correlation between feeling like crying and pre-expectation of considering oneself able to understand artworks also fits the argument that advancing past the stages of assimilation and escape into an intractable situation demanding schema change requires that a perceptual task have vested interest, leading a prospective viewer to a situation not easily dismissed.

## Studies 2 and 3

At the same time, in addition to its compelling evidence for feeling like crying as an empirical measure, Study 1 also raises important questions and caveats. Chief among these is the question of how common is feeling like crying experience? While the above Study was able to show compelling evidence for crying and for its demarcative position, as noted above, Study 1 was conducted with works specifically chosen for prior crying notoriety. This raises the potential that the findings represent a special case, or that feeling like crying itself may not so easily be found in other instances. There was also an outstanding question regarding the exact nature of meaning assessment within the viewing of the art. It was argued that the sense of meaningfulness should occur with the final aesthetic stage, as was found in our analysis. This was however also argued to coincide with a switch in the mode of assessment itself—from direct mimetic/syntactic understanding, to lack of understanding in secondary control, to a new interpretation based on experience within the final aesthetic stage. While the other correlations (self-awareness, art appraisal) support this argument, nature of meaning assessment was beyond the study. Therefore, to further explore the above questions, two subsequent studies were conducted with different museums/art.

Both Studies 2 and 3 also utilized Rothko paintings, because of its above-considered non-mimetic design. However both used quite different works in terms of design and contexts, and in settings with no expectation for crying as a response. Study 2 was conducted in another room devoted solely to a series of Rothko paintings, containing seven (~1.5–3 m) works composed of orange and red rectangles on darker ground, located at the Kawamura Memorial DIC Museum in Japan (Figure 5). This study gave the opportunity, by using the same type of abstract art within a different culture and language, to look for cross-cultural similarity in responses as they might pertain to the underlying cognitive progression. It also afforded a different color pallet, while possible contamination from viewer prior knowledge of the art/artist, although not found to be a factor above, was also further reduced. Where an argument might be made for the unique impact of the architecture/setting in the above study, the Kawamura room at the time of this study^3^ was also a truly “neutral” gallery—composed of a rectangular space with off-white walls, a central bench, no contextual information, and the paintings arranged on all four walls—situated within the larger museum. Therefore, while affording another controllable study setting, it was expected that this room and its art would be approached as just one from among many containing 20th century painting.

Study 3 was conducted during a special exhibition at London's Tate Modern. This exhibition—“Mark Rothko the Late Paintings” (Borchardt-Hume, 2008)—marked a large retrospective, including a main gallery of 14 paintings from the same series as Kawamura, with five paintings borrowed from the museum. It also included several other series and color combinations spread over nine rooms. While the rooms containing the art again had no labels, the exhibition also included two rooms devoted to educational information regarding the artist and technical aspects of his painting. This location therefore offered the chance to explore the above theory in a more general museum-wide setting, and was included primarily to test whether FLC might even be recorded in such a context.

### Participants

The Kawamura study included 30 participants (*M* age = 42; 21 female), all native Japanese. The Tate study involved 28 participants (*M* age = 53.3; 16 female) all Western European, and comfortable in English. Participants in both studies were recruited on site from the population of—unlike Study 1—paying patrons, with no offer of compensation.

### Materials and procedure

Procedure for Kawamura followed Study 1. Viewers were approached upon entering the museum. After completing a pre-test, they were instructed to engage with the targeted art. Unlike Study 1, the target room was located within a larger museum. Therefore, viewers were asked to proceed directly to this gallery. When finished they were asked to exit through another doorway at the back of this room leading directly to the lobby where they were given the post-test. Therefore, the target art was presumably the first and only art seriously encountered. In the Tate study, the pre-test was omitted. Viewers instead entered the gallery—again spatially separated from the other museum areas—without prior knowledge that they would be questioned. This afforded the opportunity to compare results from an experience without any potential priming that might be engendered by a participant's known study participation. Upon exiting through a different door viewers were met by a researcher who administered the post-test.

Questionnaires followed Study 1. However both subsequent studies made use of a paired-down version omitting several semantic scales and emotional factors which had not shown significance or which had been judged to be redundant. A new section on the post-test asking viewers to write a short answer to the question “what did the art mean?” was also added in order to ascertain mode of meaning assessment. In the Kawamura study, questions were translated into Japanese by the author and checked by two native speakers. Adjective pairs followed translation by Tanaka et al. (1963).

## Results and discussion: studies 2 and 3

Studies 2 and 3 showed general correspondence to Study 1, with 36% noting feeling like crying in both Kawamura (*M* = 3.6, *SD* = 1.9) and Tate (*M* = 3.5, *SD* = 2.1). Correlation analyses (reported for both studies in Table 2) also showed the same basic patterns as in the previous study. In Kawamura there was significant tie between FLC and *self-awareness* as well as *feeling of being watched*, a potential trigger, *epiphany*, and *understanding the artist intention*. In the Tate study, FLC showed positive correlation with *self-awareness, epiphany* and *understanding intention*.

The same hypothesized patterns were further supported when comparing between viewers reporting different combinations of confusion and FLC. As shown in Table 4, in Kawamura, unlike the first study where viewers were essentially either confused or not, results showed a high incidence of reported confusion across all viewers and split between those individuals reporting confusion without eventual FLC (*n* = 16) and those reporting both (*n* = 9). On the other hand, there was low incidence of individuals reporting neither factor (*n* = 3) and again few (*n* = 2) reporting confusion-free crying. Once again, in comparison between the two groups where confusion was reported, the magnitude was similar, suggesting the same arousal of discrepancy within the earlier stages of their respective experiences. In turn, when comparing between the two confusion outcomes, it was again those who did feel like crying who reported terms suggesting movement to insight/aesthetic experience. The “FLC” group had significantly higher *awareness of being watched* (*U* = 23.0, *p* = 0.004). The “FLC” group also showed significantly higher *epiphany* (*U* = 34.5, *p* = 0.02). There was also a non-significant trend of higher self-awareness among the “FLC” (*Mdn* = 4.0) vs. “confusion, no-FLC” group (*Mdn* = 1.0; *U* = 43.5, *p* = 0.09). (Note, “no-confusion, no-FLC” group was dropped from analysis due to small sample. However, it followed the same trend as the other studies. Results including this group are provided in Table 4).

In the Tate study, much like Study 1, division of viewers showed almost equal separation into the three posited outcomes, with 32% (*n* = 9) reporting neither confusion nor FLC, 32% (*n* = 9) reporting confusion without FLC and 32% (*n* = 9) reporting confusion and FLC, and again only one individual reporting confusion free crying. Comparison between the three main groups (Table 4) again showed equally high levels of confusion in both the “confusion, no-FLC” and “confusion, FLC” outcomes. In turn, there was also significant differences in reported *self-awareness*. as well as in the potential triggering feeling of *awareness of others/being watched*, and in reported *happiness* and *epiphany*, with *post-hoc* analysis of these latter two factors showing the FLC outcome significantly higher than both the facile and abortive conclusions.

Once again, the above patterns were even clearer in simple comparison between FLC and no-FLC subgroups. As shown in Table 5 (also Figure 7) both Tate and Study 1 showed significantly higher *need to leave*. Studies 2 and 3 showed significantly higher *awareness of others/being watched*; and all three studies showing significantly higher *epiphany*. In Kawamura, mirroring Study 1, those reporting FLC also spent roughly twice as long in the gallery (*Mdn* = 7.5 vs. 3.0 min), in this case reaching significance (*U* = 31.0, *p* = 0.03, *r* = 0.4, *Mdn* difference estimate = 4 min. [0–8]).

Correspondence to Study 1 also carried into artwork evaluation (Table 3). The Kawamura study showed significant correlations between FLC and finding the art more *intimate* and *unique*. The Tate study showed correlation with more *deep, lively* and *sincere*. Each of which typically relates to potency and activity. Reading from the opposite direction, this also meant that low FLC was concomitant with assessment of the art as more remote, common, insincere and shallow, which may be interpreted as indicating self-protection. FLC also correlated to higher ratings with the typically hedonic scales of *good* and *beautiful* in both studies, as well as—mirroring Study 1—finding the art more *meaningful*.

Regarding the specific assessment of meaning, written answers to the question “what did the art mean?” were divided into the three categories posited above: “semiotic/formal” (answers which noted the historical or mimetic qualities); “no understanding/meaninglessness;” and “experiential” (answers which equated meaning to viewing experience). In Kawamura (Table 6), Fisher's Exact test revealed significant difference (*p* = 0.03). Supporting the above hypothesis, 100% of viewers who reported FLC explained meaning as tied to personal experience—for example “looking into my inner self.” On the other hand, among the no-FLC group, 43% explained the art in experiential terms, 14% used semiotic/formal terms, and 43% said it was meaningless. In Tate, though not reaching significance (*p* = 0.09), 71% of those who felt like crying showed experiential appraisals, zero used formal/semantic assessment and the remainder reported that the art was meaningless. Among those who did not feel like crying, equal amounts (36%) reported formal/mimetic meaning or meaninglessness, and 28.5% used an experiential assessment.

**Table 6:** **Answers to “what was the meaning of the art?” organized by reported feeling like crying and type of meaning appraisal: Rothko Room, Kawamura DIC Museum, Japan**.

	**Experiential meaning**	**Semiotic/formal meaning**	**No meaning/could not understand**
**Felt like crying**	***n* = 8 (100%)**	***n* = 0 (0%)**	***n* = 0 (0%)**
	• A feeling of being sandwiched and embraced between many paintings		
	• Make the fine arts familiar		
	• Room for easing		
	• The slow experience of a movement of feeling		
	• Intuition…(after that) looking into my inner self from the effect of time		
	• Assimilation of the space and your consciousness, and a deepening		
	• At that time, your thoughts change		
	• Depends on the person		
**Did not feel like crying**	***n* = 6 (43%)**	***n* = 2 (14%)**	***n* = 6 (43%)**
	• A feeling of searching for (the essence) of art	• I understand the (historical) meaning, but I don't like it.^a^	• I don't know
	• Soak in the art and look into your own soul		
	• Face yourself…space…it's difficult	• Whatever the artist wanted the meaning to be	• I don't think they have a particular meaning
	• For me, it depends on the time of day and your own status. Every time I enter the painting to which I attend changes		• The meaning was so deep I couldn't understand
	• The room reduces you back to a neutral state		• I don't know
	• To make you think		• ?^b^
			• ?^b^

*Answers translated from original Japanese by the author. Fisher's Exact Test for differences in distribution (two-tailed), p = 0.03*.

a *Subject referred to the historical meaning in an earlier survey question and was referencing that in their answer here*.

b *Subjects wrote a literal question mark as their answer*.

Finally, in Kawamura where both pre- and post-tests were administered, self re-evaluation among those feeling like crying was again not significant. As in Study 1, there was a trend among FLC-viewers of evaluation as more *simple* (*Z* = −1.86, *p* = 0.06, *Mdn* change = 0.5 [0, 1]) and *insincere* (*Z* = −1.67, *p* = 0.09, *Mdn* change = 0.5 [0, 1]) following the art viewing. These measures are again typically tied to potency. On the other hand, once again those who reported no FLC showed significant self re-evaluations as more *humorous* (*Mdn* change = 0.0 [0,0.5], *Z* = −1.89, *p* = 0.05). This suggests that the specific humorous measure may play an intriguing role in especially abortive or non-fulfilling outcomes, marking another intriguing area for future research. In pre-expectations, Kawamura study results were non-significant. Again, no significant connections were found regarding demographics or knowledge of art or artist.

## Conclusion

This paper connected cognitive discussions of crying to the case of tears in art, considering arguments for a mechanism involving discrepancy, self-appraisal and schema change which may underlie crying and more importantly may have a fundamental similarity with insight or “aesthetic” experience as they are argued to occur within recent psychological discussion. Given this parallel, it was argued that tears—or even “feeling like” crying—may suggest important outcomes in emotion, cognition and evaluation, and may serve as a potential means of demarcating perceptual (or art) experience. Feeling like crying was hypothesized to coincide with a specific emotional progression, as well as with an “experiential” interpretation of art meaning rather than semiotic or formal; with beauty and worth rather than ugliness; with potency of art rather than devaluation, and ultimately with relative schema-change or “self-transformation.”

Three exploratory studies, in three different settings, supported most of these hypotheses, with 43% of viewers in the Houston study, 36% in the Kawamura Museum in Japan and 36% in London's Tate Modern reporting incidence of “feeling like crying” within their art experiences. Significant correlations were found between feeling like crying and emotional factors argued to correspond to full progression of this model, and with specific connection between crying and confusion, self-awareness and epiphany, argued to be the key elements demarcating the movement through the three main outcomes posited for perception, and with the latter two occurring only in cases of insight/“aesthetic” experience. Significant correlations were also between feeling like crying and appraisal of art as potent, active, meaningful, and hedonically beautiful/good. In Kawamura and Tate, where this question was considered, we also found significant distribution of “experience-based” assessment of meaning rather than direct informational or no understanding among those reporting feeling like crying.

Transversely, for those who did not feel like crying, experience omitted final pleasurable, self-reflective and insightful emotion. Non-criers also showed significant correlation to evaluations of ugliness, meaninglessness, badness of art, and surface or meaningless evaluations of the art's significance, as well as devaluation of the self as more humorous—suggesting the potential importance of feeling like crying when considered within an empirical context. Perhaps equally intriguing, our basic ability to record meaningful answers to the question “did you feel like crying”—occurring in two settings with no prior reason to expect such reaction and, in the case of Study 3, in a setting in which individuals had wandered a gallery of multiple artworks—coupled with the consistency of findings when individuals did answer affirmatively or negatively to this question, and with cross-culture consistency, suggests “feeling like crying” might be a common element in art experience.

This study also of course comes with caveats and demands for future research. The studies are largely correlational. While the connections between responses and feeling like crying, as well as the differences when viewers either do or do not report this outcome, support the hypothesized progression, this must be substantiated in further studies, especially also with larger samples and a broader range of art. This would also allow for more advanced statistical modeling (e.g., multilevel models), which would permit better causative assessment and consideration of interactions between individual elements. In addition, the artwork and self evaluations would benefit from factor analyses in order to better test the implications of the data. In fact, as noted above, I suggest that this paper be considered as a first basis for such future research, and encourage the reader to consider the findings more for their qualitative suggestion of factor relationships that could be tested in further work. Future study should also test for posited change in the self image, which here did not reach significance, and might also further develop the topic through a combination of quantitative and qualitative approaches. Finally, it must be stressed that this paper does not attempt to “prove” the cognitive theory of crying, which is beyond the scope of the present study. In the attempt to study artworks in their “natural” environment I also did not verify tears in the gallery. Although supported by previous research, I make no attempt to assert whether “feeling like crying” corresponds to physical tears, or whether this too might show intriguing difference.

That said, this paper raises the tentative, and compelling, conclusion that answering in the affirmative to “did you feel like crying” may indeed be a salient indicator of outcome in art or other perceptual experience.

## Author and contributions

All aspects of the study, including conception, implementation, analysis and writing were led by MP.

### Conflict of interest statement

The author declares that the research was conducted in the absence of any commercial or financial relationships that could be construed as a potential conflict of interest.

## Footnotes

^1^Put by Sartre, more poetically, and touching on the forthcoming argument in art: in crying the individual “changes [their] relation to the world in order that the world may change its qualities” (in Lutz, p. 240).

^2^Although outside the scope of this paper, in evolutionary discussion there is speculation that would connect function of crying precisely to protection of the individual from distractions impeding an “aesthetic” phase. Crying may signal to unfamiliar others that one should be left alone, or, for intimate others crying may signal need for support while one withdraws and re-evaluates (Neale, 1986; Hill and Martin, 1997). Alternatively, Barbalet (2005, p. 139) frames crying directly in a personal context, contending that the “function of weeping [is] to communicate to the self an incident of transformation,” and acting to steer individuals away from other obligations thereby reinforcing cognitive processes of reflection/re-organization.

^3^This arrangement, although keeping the same artworks, was moved to a larger gallery in 2007.
